# Supplementary material for: Strong pathogen competition in neonatal gut colonisation
Source: Nat Commun. 2022 Dec 1;13:7417. doi: 10.1038/s41467-022-35178-5 (PMC9715557; doi:10.1038/s41467-022-35178-5)
Supplement: Supplementary file 3 — Description to Additional Supplementary Information [file 41467_2022_35178_MOESM3_ESM.pdf]

Description of Additional Supplementary Files

Supplementary Data 1 contains information about the Enterococcus faecalis strains analysed in the manuscript that were identified as harbouring antibiotic resistance-conferring genes to major antibiotic classes

Supplementary Data 2 contains the list of identified lineages (bins) in each sample that received high or very high score (1 or 2) from demix_check. These bins are called reliable identifications in the manuscript.
