# Supplementary material for: Strong pathogen competition in neonatal gut colonisation
Source: Nat Commun. 2022 Dec 1;13:7417. doi: 10.1038/s41467-022-35178-5 (PMC9715557; doi:10.1038/s41467-022-35178-5)
Supplement: Supplementary file 6 — Reporting Summary [file 41467_2022_35178_MOESM6_ESM.pdf]

nature portfolio

Corresponding author(s): Tommi Mäklin and Jukka Corander

Last updated by author(s): 16-11-2022

Reporting Summary

Nature Portfolio wishes to improve the reproducibility of the work that we publish. This form provides structure for consistency and transparency in reporting. For further information on Nature Portfolio policies, see our Editorial Policies and the Editorial Policy Checklist.

Statistics

For all statistical analyses, confirm that the following items are present in the figure legend, table legend, main text, or Methods section.

n/a	Confirmed	
☐	☒	The exact sample size (*n*) for each experimental group/condition, given as a discrete number and unit of measurement
☐	☒	A statement on whether measurements were taken from distinct samples or whether the same sample was measured repeatedly
☐	☒	The statistical test(s) used AND whether they are one- or two-sided<br>*Only common tests should be described solely by name; describe more complex techniques in the Methods section.*
☒	☐	A description of all covariates tested
☐	☒	A description of any assumptions or corrections, such as tests of normality and adjustment for multiple comparisons
☐	☒	A full description of the statistical parameters including central tendency (e.g. means) or other basic estimates (e.g. regression coefficient) AND variation (e.g. standard deviation) or associated estimates of uncertainty (e.g. confidence intervals)
☐	☒	For null hypothesis testing, the test statistic (e.g. *F*, *t*, *r*) with confidence intervals, effect sizes, degrees of freedom and *P* value noted<br>*Give P values as exact values whenever suitable.*
☒	☐	For Bayesian analysis, information on the choice of priors and Markov chain Monte Carlo settings
☒	☐	For hierarchical and complex designs, identification of the appropriate level for tests and full reporting of outcomes
☒	☐	Estimates of effect sizes (e.g. Cohen's *d*, Pearson's *r*), indicating how they were calculated

Our web collection on statistics for biologists contains articles on many of the points above.

Software and code

Policy information about availability of computer code

Data collection

No software were used to collect the data.

Data analysis

The following software were used to analyse the data:
- MetaPhlAn v3.0
- Themisto v2.1.0
- PopPUNK v2.4.0 and v2.2.0
- mSWEEP v1.6.0
- mGEMS v1.2.0
- demix_check (commit 18470d3)
- fastp v0.23.1
- shovill v1.1.0
- FastSpar v1.0.0
- SKA v1.0.0
- fastMLST v0.0.15
- AMRFinderPlus v3.10.18
- R v4.2.0 and v4.0.5
- Kleborate v2.1.0
- UpSetR package v1.4.0

Custom scripts used in the study are available from GitHub: https://github.com/tmaklin/baby-microbiome-paper-plots

No commercial software or unpublished custom software were used.

For manuscripts utilizing custom algorithms or software that are central to the research but not yet described in published literature, software must be made available to editors and reviewers. We strongly encourage code deposition in a community repository (e.g. GitHub). See the Nature Portfolio guidelines for submitting code & software for further information.

Data

Policy information about availability of data

All manuscripts must include a data availability statement. This statement should provide the following information, where applicable:

- Accession codes, unique identifiers, or web links for publicly available datasets
- A description of any restrictions on data availability
- For clinical datasets or third party data, please ensure that the statement adheres to our policy

The species-level pseudoalignment index for Themisto v2.1.0, the E. coli, E. faecalis, and Klebsiella species-specific indexes are all available at Zenodo (species-level index doi: 10.5281/zenodo.6656881, E. coli index doi: 10.5281/zenodo.6656897, E. faecalis index: 10.5281/zenodo.6656903, Klebsiella index: 10.5281/zenodo.6656911). Results from the mGEMS pipeline which were assigned high or very high confidence scores using demix_check, forming the core of the analyses presented, are listed in Supplementary Data 2. For all figures presented in the manuscript, source data are provided as a Source Data file. The sequencing data used in this study are available in the European Nucleotide Archive under accession codes ERP115334 (wholegenome shotgun metagenomics sequencing data) and ERP024601 (isolate sequencing data).

Human research participants

Policy information about studies involving human research participants and Sex and Gender in Research.

Reporting on sex and gender	Information about the sex of the research participants was collected in the source study for the sequencing data, and is described there (https://www.nature.com/articles/s41586-019-1560-1). We did not use information about sex/gender in our study as it was not relevant to the research question.
Population characteristics	The population characteristics are described in the study that was the source of the analysed sequencing data (https://www.nature.com/articles/s41586-019-1560-1).
Recruitment	Recruitment of the study participants is described in the source study for the sequencing data (https://www.nature.com/articles/s41586-019-1560-1).
Ethics oversight	The source study for the sequencing data was approved by the NHS London – City and East Research Ethics Committee (REC reference 12/LO/1492) (See https://www.nature.com/articles/s41586-019-1560-1, section "Study population").

Note that full information on the approval of the study protocol must also be provided in the manuscript.

Field-specific reporting

Please select the one below that is the best fit for your research. If you are not sure, read the appropriate sections before making your selection.

☒ Life sciences ☐ Behavioural & social sciences ☐ Ecological, evolutionary & environmental sciences

For a reference copy of the document with all sections, see nature.com/documents/nr-reporting-summary-flat.pdf

Life sciences study design

All studies must disclose on these points even when the disclosure is negative.

Sample size	We chose the particular neonatal cohort for multiple reasons. Firstly, because of its longitudinal aspect which enabled us to investigate dynamics of the competition. Secondly, due to its high sequencing depth and state-of-the-art short-read technology used, which enabled us to assemble the genomes of the colonising strains in sufficient detail for genomic epidemiological analysis. Thirdly, the DNA obtained from a sufficiently large number of faecal samples via the culture-independent approach used in the study provided an opportunity for us to screen for the pathogens in an unbiased and robust manner. Finally, the neonatal cohort was ideal for the study since it enabled us to probe the competition dynamics between the pathogen strains and species when they colonise a niche that is approximately empty, unlike the situation later in life. In addition, we screened a large body of microbiome literature to identify other published studies that would be suitable for our purposes, but only the chosen study (www.nature.com/articles/s41586-019-1560-1) was deemed appropriate, satisfying all these desiderata.
Data exclusions	Data were not excluded.
Replication	Not applicable, data were obtained from a previously published collection (https://www.nature.com/articles/s41586-019-1560-1).
Randomization	Randomization was not used because we did not attempt to estimate any treatment or causal effects, aiming instead to study and describe

Randomization	the colonisation process and competition dynamics in the neonatal cohort. Randomization is not relevant in this sort of study setting.
Blinding	Blinding was not used and was not relevant for the same reasons as randomization (see above).

Reporting for specific materials, systems and methods

We require information from authors about some types of materials, experimental systems and methods used in many studies. Here, indicate whether each material, system or method listed is relevant to your study. If you are not sure if a list item applies to your research, read the appropriate section before selecting a response.

Materials & experimental systems

n/a	Involved in the study
☒	☐ Antibodies
☒	☐ Eukaryotic cell lines
☒	☐ Palaeontology and archaeology
☒	☐ Animals and other organisms
☒	☐ Clinical data
☒	☐ Dual use research of concern

Methods

n/a	Involved in the study
☒	☐ ChIP-seq
☒	☐ Flow cytometry
☒	☐ MRI-based neuroimaging
